# Chan-Chuang and resistance exercise for drug rehabilitation: a randomized controlled trial among Chinese male methamphetamine users

**DOI:** 10.3389/fpubh.2023.1180503

**Published:** 2023-10-26

**Authors:** Hansen Li, Chao Wang, Xuemei Huang, Lubing Xu, Yang Cao, Jiong Luo, Guodong Zhang

**Affiliations:** ^1^Research Center for Exercise Detoxification, College of Physical Education, Southwest University, Chongqing, China; ^2^Chongqing Xishanping Education and Correction Center, Chongqing, China; ^3^Clinical Epidemiology and Biostatistics, School of Medical Sciences, Faculty of Medicine and Health, Örebro University, Örebro, Sweden; ^4^Unit of Integrative Epidemiology, Institute of Environmental Medicine, Karolinska Institutet, Stockholm, Sweden

**Keywords:** Chan-Chuang, mindfulness, strength training, methamphetamine, qigong, drug

## Abstract

**Objective:**

To examine the health benefits of Chan-Chuang and resistance exercise.

**Methods:**

We deployed an 8-week randomized controlled trial, in which 76 male methamphetamine users were allocated to control (*n* = 25), Chan-Chuang (*n* = 26), and residence exercise groups (*n* = 25). Our primary outcomes were drug craving, mental wellbeing, sleep quality, heart rate (HR), systolic blood pressure (SBP), diastolic blood pressure (DBP), and mean arterial pressure (MAP). Our secondary outcomes were body mass index (BMI), vital capacity, grip strength, balance, and vertical jump.

**Results:**

Chan-Chuang exercise resulted in reduced HR, DBP, and MAP, along with improvements in vital capacity, grip strength, and balance compared to the control group. Resistance exercise reduced SBP and MAP, and also improved vital capacity, grip strength, balance, and vertical jump.

**Conclusion:**

These findings may support the role of Chan-Chuang and resistance exercise in maintaining the physical fitness of methamphetamine users at mandatory detention centers.

## 1. Introduction

Drug consumption is a global problem that has caused over 200,000 fatalities every year (1). The Report on the Drug Situation in China in 2020 has disclosed that there are more than 1.8 million illegal drug users in China, and over 57% of them primarily use synthetic drugs (2). Methamphetamine is reported to be the most consumed synthetic drug (3) that results in many serious adverse consequences (4). Therefore, China has been taking a punitive and hard-line strategy toward drug use and trafficking. For example, captured drug users are usually sent to mandatory detention centers for correctional education and detoxification (3).

Among numerous drug rehabilitation strategies, physical exercise is a promising one that may reduce depression, toxicity in the brain, and the chance of drug relapse (5, 6). Nevertheless, there is still little understanding of how the type of physical exercise can alter health outcomes among drug users, as existing studies usually employ combined exercise interventions. Moreover, some exercise types, such as isometric exercise, have been rarely studied. Theoretically, isometric exercise is a mild exercise that can lower blood pressure and improve strength (7, 8). Importantly, isometric exercise hardly relies on equipment and is therefore suitable for detained people.

China has explored many featured drug rehabilitation treatments, such as herbal therapy and acupuncture (9). There is a traditional Chinese Qigong called Chang-Chuang, which is an isometric exercise characterized by mindfulness. Some scholars describe it as a “mind–body exercise” that combines meditation and physical exercise (10), which can promote the physical and mental health of critically ill patients (11–13). Given the fact that drug users usually have poor health conditions, Chan-Chuang may also play some roles in their health promotion. Therefore, we designed this study to preliminarily examine the effects of Chan-Chuang and resistance training on the rehabilitation of Chinses methamphetamine users.

## 2. Materials and methods

### 2.1. Trial design

This study was a randomized controlled trial with a parallel design, including two experimental groups and a control group, with an allocation ratio of 1:1:1. We offered 100 renminbi (RMB) awards per week to encourage participation. The study protocol has been registered in the Chinese Clinical Trial Registry under the registration. Recruited participants were allowed to withdraw or miss part of the exercise or test. However, one would be disqualified and excluded from the analysis if he had been absent for three consecutive exercises.

### 2.2. Participants

Our participants were recruited from a drug rehabilitation center for males only in Chongqing, China. Inclusion criteria were males aged between 20 and 50 years and methamphetamine users (mainly used methamphetamine and were detained due to its consumption). Exclusion criteria were having regular exercise habits (to avoid additional exercise masking or amplifying the true effects of the intervention), having physical disabilities or other serious symptoms known to affect mobility, and having difficulties in following the intervention actions. This study involving human participants was reviewed and approved by the Institutional Review Board of the College of Physical Education of Southwest University. Participation in this study was purely voluntary. We introduced our plan and distributed recruitment messages to the detained people through routine meetings at the correctional center. Written informed consent to participate in this study was provided by our participants.

### 2.3. Intervention

**Figure 1:**
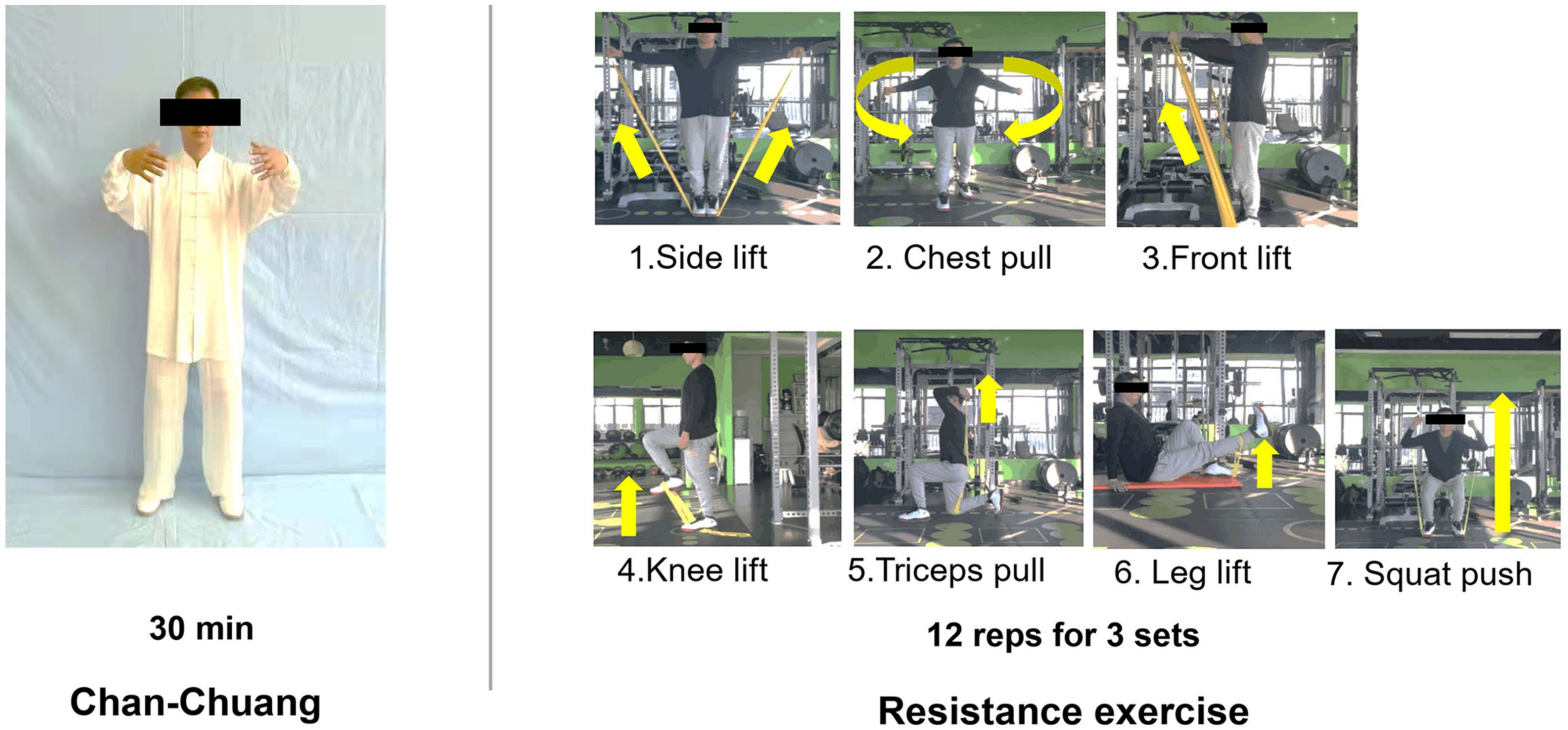
Intervention and designed exercise.

The control group received no extra intervention and continued their routine. The Chan-Chuang and resistance exercise groups were guided to perform the corresponding exercises in the following eight weeks, and their routines were retained. The two groups practiced in two different activity rooms at the detention center, and our researchers were on hand to provide guidance and supervision during the exercises. The participants carried out the assigned exercise at 10 a.m. from Monday to Friday and underwent weekly tests every weekend throughout the intervention. Each exercise lasted 30–40 min (including a 5-min warm-up), and their detailed exercise contents are demonstrated in Figure 1. Based on the recommended exercise prescriptions for Chinese drug users (14), we designed a moderate-intensity (50–60% of perceived exertion) resistance exercise using elastic bands. The specific exercises and repetition setting for resistance exercise can be found in Figure 1. Chan-Chuang is an isometric exercise. Participants maintained a posture with their knees slightly bent and their arms raised to chest height (as indicated in Figure 1). This position was held for a required duration.

During the intervention, if participants failed to adhere to our research protocol, we would assess the degree of non-compliance, engage in communication with participants to resolve the issue, offer support and encouragement, and carefully record interactions and the underlying reasons. During data collection and analysis, we would consider the presence of non-adherent participants and utilize sensitivity analysis and statistical adjustments as necessary. Meanwhile, we would uphold ethical standards throughout the entire research process and maintain transparent reporting of any instances of non-compliance.

### 2.4. Sample size

After the assessment for eligibility, a total of 71 participants were recruited. A power calculation was conducted before the interventions using G-power software. The effect size utilized in the calculation was set at 0.30 (15), and the significance level α was set at 0.05. The achieved statistical power level regarding our primary outcomes (which have been repeatedly tested for 8 times) was 0.87. However, the achieved statistical power level regarding our secondary outcomes was only 0.33.

### 2.5. Outcomes

There were primary and secondary outcomes. The primary outcomes were measured before (at the baseline) and every weekend throughout the intervention. The secondary outcomes were measured only before and after the intervention.

#### 2.5.1. Primary outcomes

Our primary outcomes included drug craving, mental wellbeing, sleep quality, heart rate (HR), systolic blood pressure (SBP), diastolic blood pressure (DBP), and mean arterial pressure (MAP). These outcomes were measured at 9 a.m. every Saturday (fasting state) in the detention center’s classrooms.

1. Drug craving: A Visual Analog Scale (VAS) scale was employed for self-reports, which is a common tool for measuring drug craving levels (16).
2. Mental wellbeing: The WHO-5 wellbeing index (17, 18) was employed in this study, which showed an acceptable internal consistency in our baseline test (Cronbach’s alpha >0.7).
3. Sleep quality: The Pittsburgh Sleep Quality Index (PSQI) was employed to indicate sleep quality (19, 20). A higher total score indicates worse sleep quality. The PSQI scale showed an acceptable internal consistency in our baseline test (Cronbach’s alpha >0.7)
4. HR/SBP/DBP/MAP: Heart rate and blood pressure were measured by portable monitors (instrument model: OMRON U10L).
5. All the self-reported scales were obtained under a timeframe of “the past week.”

#### 2.5.2. Secondary outcomes

The secondary outcomes were measured at the baseline and the last weekend of the intervention (between 2 and 5 p.m.) in the detention center’s physical examination room. Our primary outcomes were measured by the following means:

1. Body mass index (BMI): BMI is defined as the body mass (kilogram) divided by the square of the body height.
2. Vital capacity: Vital capacity refers to the total amount of air exhaled after maximal inhalation, which is often used as an index to evaluate general health.
3. Grip strength: Grip strength (in N) is an indicator of the general level of human upper limb strength.
4. Balance: Keeping balance is an essential ability to avoid falls and other injuries. Participants were guided to stand on one foot with their eyes closed, and their duration/time of successfully maintaining balance (in seconds) was recorded by a ground sensor.
5. Vertical jump: Vertical jump is a common predictor of health-related fitness performance (21). Our participants were asked to perform a maximum voluntary jump on a ground sensor. The height was calculated based on the pressure detected by the ground sensor.

### 2.6. Randomization

This was a simple randomization (without blocking) and the random allocation sequence was obtained by drawing lots with pieces of paper. Such a method is straightforward and relies on chance. Specifically, we placed pieces of paper with participant names into an opaque box, each piece of paper of equal size. After multiple shuffles, we had an experimenter randomly draw out one piece of paper at a time and place it in positions symbolizing the three groups. The randomization, allocation, intervention, and data collection were all conducted by the same research team members.

### 2.7. Blinding

The characteristics of the exercise intervention make it difficult to implement a completely blind approach (22, 23). We did not inform participants about the group assignments and conducted interventions and tests separately. However, participants could interact with each other outside of interventions and might be aware of our grouping design. Moreover, the same group of experiment staff members participated in intervention guidance as well as data collection and analysis. Therefore, it can be considered that this study did not employ blinding methods.

### 2.8. Analyses

Prior to the intervention, a baseline test was performed for the three groups and any initial differences between the groups were subsequently controlled for in regression analyses. Since the outcomes did not follow a normal distribution, we employed the Kruskal-Wallis test for comparison.

Regarding our primary outcomes (drug craving, mental health, sleep problem, HR, SBP, DBP, and MAP), we used the Generalized Estimating Equation (GEE) to compare outcomes among groups. The GEEs are widely used to analyze interconnected longitudinal data with a non-normal distribution or missing data (24, 25). Since GEE can cope with missing values without excluding samples (26), we did not impute missing values in the dataset and reported the number of samples and observations that have been used in the relevant analyses. We constructed two models. In the first model, group setting and time (measured as the number of weeks from baseline) were treated as factors to test their main effect, meanwhile, the main effect of the “group-by-time” term was also tested (27). This is because the effect of the interaction term was found statistically significant in our analyses. In the subsequent analyses for simple effects, the post-hoc pairwise comparisons among groups at different time points were performed based on the interaction term. In the second model, we further included baseline assessments as covariates for adjustment because we detected some imbalances at our baseline. In these analyses, we used an independent working correlation matrix for the analysis as this matrix resulted in the lowest QIC levels (28, 29).

As our secondary outcomes (BMI, vital capacity, grip strength, balance, vertical jump) were assessed solely at baseline and after our intervention, we applied the Generalized Linear Model (GLM) to compare these outcomes among groups. Following the method of GEE, we constructed two models. One is a crude model where only the main effect of the group setting was tested. In the other model, we incorporated corresponding baseline measurements as covariates to account for potential differences at baseline (30, 31).

In the end, all our participants successfully completed the study, adhering to our instructions as anticipated. Therefore, our analyses are both intention-to-treat analysis and per-protocol analysis.

### 2.9. Sensitivity analysis

We also conducted an additional analysis that incorporated subjects’ characteristics (including age, drug use history, and detention duration) as covariates into the GEE model and re-evaluated the main effect of the intervention on our primary outcome variables. The value of *p* was adjusted with Bonferroni correction when performing multiple comparisons, and a value of *p* of smaller than 0.05 was considered statistically significant. The analyses were implemented using IBM SPSS Statistics 25.0 (SPSS Inc., Chicago, IL, United States).

## 3. Results

### 3.1. Baseline analysis

The characteristics of the participants at the baseline are shown in Supplementary Table S1. Our participants were at the age of around 34 years old, had used methamphetamine for around 74 months, and had been kept in the mandatory detention center for around 9 months on average. The Kruskal-Wallis test revealed significant differences in drug craving, mental wellbeing, DBP, MAP, and BMI among groups at the baseline (*p* < 0.05).

### 3.2. The main effects of experimental settings on the primary and secondary outcomes

Table 1 demonstrates the mean values of each group and the mean differences between the intervention groups and the control group (evaluated by the GEE models). The detailed pairwise comparisons among the three groups can be found in Supplementary Table S2.

**Table 1:** Outcomes and pairwise comparisons between the groups.

Outcome	Group	Participants	Observations	Crude model		Adjusted model	
				Mean (95%CI)	Mean difference (95%CI)	Mean (95%CI)	Mean difference (95%CI)
Drug craving	Control	25	182	1.65 (0.44, 2.85)	Referent	1.70 (0.90, 2.49)	Referent
	Chan-Chuang	26	205	2.46 (1.48, 3.44)	0.81 (−1.09, 2.70)	2.00 (1.06, 2.93)	0.30 (−1.28, 1.87)
	Resistance	25	197	0.34 (0.07, 0.61)	−1.31 (−2.82, 0.20)	0.78 (0.35, 1.20)	−0.92 (−1.89, 0.05)
Mental wellbeing	Control	25	200	15.57 (12.77, 18.37)	Referent	16.29 (14.77, 17.81)	Referent
	Chan-Chuang	26	206	19.59 (17.43, 21.74)	4.02 (−0.3, 8.34)	16.83 (14.77, 18.89)	0.54 (−2.63, 3.71)
	Resistance	25	197	14.66 (12.76, 16.55)	−0.92 (−5.04, 3.22)	16.81 (16.29, 17.34)	0.52 (−1.45, 2.49)
Poor sleep quality	Control	25	200	12.48 (9.81, 15.14)	Referent	12.40 (10.60, 14.21)	Referent
	Chan-Chuang	26	206	14.11 (11.55, 16.67)	1.63 (−2.88, 6.15)	11.90 (10.12, 13.68)	−0.51 (−3.59, 2.58)
	Resistance	25	197	10.09 (7.78, 12.40)	−2.39 (−6.7, 1.93)	12.48 (11.86, 13.09)	0.07 (−2.27, 2.41)
HR	Control	25	197	78.49 (76.23, 80.75)	Referent	79.28 (77.17, 81.39)	Referent
	Chan-Chuang	26	180	74.22 (71.56, 76.88)	−4.27 (−8.53, 0.00)	75.36 (73.07, 77.64)	−3.92 (−7.72, −0.13)
	Resistance	25	194	78.98 (76.51, 81.45)	0.49 (−3.59, 4.58)	77.40 (76.18, 78.62)	−1.88 (−4.82, 1.06)
SBP	Control	25	199	124.69 (120.89, 128.50)	Referent	122.89 (120.74, 125.04)	Referent
	Chan-Chuang	26	179	120.61 (119.21, 122.02)	−4.08 (−9.03, 0.88)	122.68 (120.9, 124.46)	−0.21 (−3.62, 3.20)
	Resistance	25	194	119.1 (117.53, 120.67)	−5.59 (−10.62, −0.57)	118.29 (117.55, 119.03)	−4.60 (−7.39, −1.81)
DBP	Control	25	198	84.14 (81.33, 86.96)	Referent	83.76 (81.34, 86.17)	Referent
	Chan-Chuang	26	178	74.45 (72.63, 76.27)	−9.69 (−13.79, −5.60)	75.06 (72.53, 77.59)	−8.69 (−13.21, −4.18)
	Resistance	25	181	80.92 (79.15, 82.68)	−3.23 (−7.29, 0.83)	80.69 (79.29, 82.09)	−3.07 (−6.43, 0.30)
MAP	Control	25	198	97.64 (94.68, 100.60)	Referent	96.93 (94.73, 99.13)	Referent
	Chan-Chuang	26	177	89.48 (87.98, 90.97)	−8.16 (−12.22, −4.11)	90.60 (88.06, 93.14)	−6.33 (−10.77, −1.89)
	Resistance	25	181	93.75 (92.22, 95.28)	−3.89 (−7.96, 0.19)	93.34 (92.24, 94.44)	−3.59 (−6.53, −0.64)
BMI	Control	25	25	24.91 (23.87, 25.95)	Referent	24.17 (23.87, 24.47)	Referent
	Chan-Chuang	24	24	23.33 (22.07, 24.59)	−1.58 (−3.57, 0.41)	24.57 (24.19, 24.96)	0.40 (−0.21, 1.02)
	Resistance	16	16	24.31 (23.26, 25.37)	−0.60 (−2.41, 1.21)	24.01 (23.71, 24.31)	−0.16 (−0.68, 0.35)
Vital capacity	Control	25	25	2566.4 (2326.3, 2806.5)	Referent	2675.36 (2506.98, 2843.74)	Referent
	Chan-Chuang	24	24	3478.11 (3195.15, 3761.07)	911.71 (458.43, 1364.99)	3391.85 (3194.61, 3589.09)	716.49 (397.4, 1035.57)
	Resistance	18	18	3,056 (2810.95, 3301.05)	489.60 (70.56, 908.64)	2997.43 (2823.43, 3171.43)	322.07 (24.85, 619.29)
Grip strength	Control	25	25	29.16 (24.88, 33.44)	Referent	28.81 (24.54, 33.07)	Referent
	Chan-Chuang	24	24	43.72 (38.68, 48.76)	14.56 (6.48, 22.63)	43.44 (38.43, 48.44)	14.63 (6.65, 22.62)
	Resistance	18	18	39.58 (35.21, 43.94)	10.42 (2.95, 17.88)	40.15 (35.74, 44.57)	11.35 (3.75, 18.95)
Balance	Control	19	19	14.23 (7.38, 21.09)	Referent	13.35 (6.62, 20.07)	Referent
	Chan-Chuang	20	20	28.48 (21.23, 35.73)	14.25 (2.07, 26.44)	29.12 (22.26, 35.98)	15.77 (3.99, 27.56)
	Resistance	17	17	25.55 (18.87, 32.23)	11.32 (−0.38, 23.01)	26.24 (19.91, 32.57)	12.89 (1.55, 24.23)
Vertical Jump	Control	22	22	32.12 (30.11, 34.14)	Referent	32.47 (30.64, 34.29)	Referent
	Chan-Chuang	24	24	35.23 (32.89, 37.57)	3.11 (−0.67, 6.88)	35.82 (33.73, 37.91)	3.36 (−0.02, 6.73)
	Resistance	17	17	36.64 (34.67, 38.61)	4.52 (1.08, 7.96)	35.89 (34.08, 37.7)	3.43 (0.27, 6.58)

The adjusted model was adjusted for baseline assessments.

In the crude model, compared to the control group, the Chan-Chuang group had lower levels of DBP (Mean difference (MD) = −9.690; 95%CI = −13.786 to −5.594; *p* < 0.001) and MAP (MD = −8.163; 95%CI = −12.217 to −4.109; *p* < 0.001), as well as higher levels of vital capacity (MD = 911.711, 95%CI = 485.434 to 1364.989; *p* < 0.001), grip strength (MD = 14.557; 95%CI = 6.480 to 22.633; *p* < 0.001), and balance (MD = 14.251; 95%CI = 2.065 to 26.437; *p* = 0.015). In the model adjusted for baseline assessments, the HR level of the Chan-Chuang group became significantly lower than that of the control group (MD = −3.922; 95%CI = −7.718 to −0.125; *p* = 0.04), while other results were not substantially changed.

In the crude model, compared to the control group, the resistance exercise group had lower levels of SBP (MD = −5.591; 95%CI = −10.617 to −0.565; *p* = 0.023), as well as higher levels of vital capacity (MD = 489.600; 95%CI = 70.557 to 908.643; *p* = 0.015), grip strength (MD = 10.415; 95%CI = 2.948 to 17.882; *p* = 0.003), and vertical jump (MD = 4.520; 95%CI = 1.077 to 7.963; *p* = 0.005). In the adjusted model, the differences in MAP (MD = −3.587; 95%CI = −6.532 to −0.642; *p* = 0.011) and balance (MD = 12.891; 95%CI = 1.552 to 24.231; *p* = 0.019) levels between the two groups became statistically significant, while the other differences were not substantially changed.

### 3.3. Differences in outcomes among groups at different timepoints

Table 2 presents the means of variables for each group in weekly tests, along with the mean differences between the intervention groups and the control group (evaluated by the GEE models). The detailed pairwise comparisons among the three groups can be found in Supplementary Table S3. All variables exhibit distinctive fluctuations over time in both the weekly measurement means and inter-group differences. Due to the pandemic issue, we lost the DBP data for the second week of the Chan-Chuang group, so the corresponding MAP was not calculated. In the adjusted model, except for the first (MD = 0.249; 95%CI = −4.592 to 5.090; *p* = 1.000) and third (MD = −2.030; 95%CI = −6.378 to 2.318; *p* = 0.791) weeks, the SBP values of the resistance exercise group were significantly lower than those of the Chan-Chuang group in all other weeks (*p* < 0.05). However, the resistance group showed higher levels of DBP than the Chan-Chuang group in the third (MD = 8.418; 95%CI = 3.896 to 12.940; *p* < 0.001), sixth (MD = 8.692; 95%CI = 3.944 to13.440; *p* < 0.001), and eighth (MD = 7.494; 95%CI = 1.230 to 13.759; *p* = 0.013) weeks.

**Table 2:** Weekly outcomes and comparisons between the groups.

Model	Outcome	Time (Week)	Control (C)		Chan-Chuang (CC)		Resistance (R)		CC vs. C	R vs. C
			Mean (95%CI)	*N*	Mean (95%CI)	*N*	Mean (95%CI)	*N*	MD (95%CI)	MD (95%CI)
Crude	Craving	1	1.56 (0.39, 2.73)	25	1.65 (0.84, 2.47)	26	0.28 (0.00, 0.56)	25	0.09 (−1.65, 1.84)	−1.28 (−2.75, 0.19)
		2	1.72 (0.56, 2.88)	25	1.81 (0.87, 2.74)	26	0.48 (0.08, 0.88)	25	0.09 (−1.73, 1.91)	−1.24 (−2.74, 0.26)
		3	1.48 (0.35, 2.61)	25	2.24 (1.24, 3.24)	25	0.56 (0.13, 0.99)	25	0.76 (−1.08, 2.60)	−0.92 (−2.39, 0.55)
		4	1.82 (0.37, 3.27)	22	3.24 (1.83, 4.65)	25	0.44 (0.00, 0.87)	23	1.42 (−1.05, 3.89)	−1.38 (−3.23, 0.47)
		5	1.90 (0.35, 3.46)	20	2.32 (1.08, 3.56)	25	0.46 (−0.05, 0.96)	24	0.42 (−2.01, 2.85)	−1.44 (−3.44, 0.55)
		6	1.16 (0.06, 2.26)	25	2.58 (1.25, 3.91)	26	0.20 (−0.05, 0.45)	25	1.42 (−0.69, 3.52)	−0.96 (−2.33, 0.41)
		7	2.00 (0.41, 3.59)	20	2.27 (1.08, 3.46)	26	0.08 (−0.07, 0.23)	25	0.27 (−2.15, 2.69)	−1.92 (−3.87, 0.03)
		8	1.55 (0.20, 2.90)	20	3.54 (2.12, 4.95)	26	0.24 (−0.08, 0.56)	25	1.99 (−0.40, 4.38)	−1.31 (−3.01, 0.39)
	Wellbeing	1	16.04 (13.02, 19.06)	25	21.00 (18.99, 23.01)	26	16.44 (13.82, 19.06)	25	4.96 (0.52, 9.40)	0.40 (−4.49, 5.29)
		2	15.28 (12.59, 17.97)	25	22.00 (19.28, 24.72)	26	15.96 (13.46, 18.47)	25	6.72 (2.05, 11.39)	0.68 (−3.81, 5.17)
		3	14.84 (12.02, 17.66)	25	18.69 (15.90, 21.49)	26	14.52 (12.16, 16.88)	25	3.85 (−1.00, 8.70)	−0.32 (−4.82, 4.18)
		4	16.08 (13.00, 19.16)	25	22.52 (19.35, 25.69)	25	14.00 (11.86, 16.14)	24	6.44 (1.04, 11.84)	−2.08 (−6.66, 2.50)
		5	15.72 (12.55, 18.89)	25	17.36 (14.54, 20.18)	25	14.92 (12.61, 17.22)	24	1.64 (−3.54, 6.82)	−0.80 (−5.59, 3.98)
		6	15.40 (12.02, 18.78)	25	17.19 (14.38, 20.01)	26	13.84 (11.86, 15.82)	25	1.79 (−3.58, 7.17)	−1.56 (−6.35, 3.23)
		7	15.36 (12.19, 18.53)	25	21.27 (18.29, 24.25)	26	13.40 (11.18, 15.62)	25	5.91 (0.60, 11.22)	−1.96 (−6.69, 2.77)
		8	15.84 (12.70, 18.98)	25	16.65 (14.23, 19.08)	26	14.17 (11.71, 16.63)	24	0.81 (−4.03, 5.66)	−1.67 (−6.55, 3.20)
	Sleep quality	1	14.64 (11.29, 17.99)	25	15.39 (12.23, 18.54)	26	11.56 (8.66, 14.46)	25	0.74 (−4.88, 6.37)	−3.08 (−8.50, 2.34)
		2	13.24 (10.50, 15.98)	25	17.23 (14.15, 20.32)	26	11.88 (8.84, 14.92)	25	3.99 (−1.05, 9.03)	−1.36 (−6.36, 3.64)
		3	12.64 (10.06, 15.22)	25	13.00 (9.89, 16.11)	26	10.56 (7.62, 13.50)	25	0.36 (−4.57, 5.29)	−2.08 (−6.86, 2.70)
		4	11.60 (9.12, 14.08)	25	15.84 (12.49, 19.19)	25	9.00 (6.41, 11.59)	24	4.24 (−0.85, 9.33)	−2.60 (−6.98, 1.78)
		5	12.84 (9.93, 15.75)	25	13.16 (9.60, 16.72)	25	10.58 (7.95, 13.21)	24	0.32 (−5.29, 5.93)	−2.26 (−7.05, 2.53)
		6	11.68 (8.84, 14.52)	25	12.96 (9.82, 16.10)	26	9.08 (6.63, 11.53)	25	1.28 (−3.89, 6.45)	−2.60 (−7.18, 1.98)
		7	11.20 (8.09, 14.31)	25	12.89 (9.39, 16.38)	26	8.92 (6.42, 11.42)	25	1.68 (−4.04, 7.41)	−2.28 (−7.16, 2.60)
		8	11.96 (8.60, 15.32)	25	12.39 (9.59, 15.18)	26	9.13 (6.65, 11.60)	24	0.42 (−4.91, 5.76)	−2.84 (−7.93, 2.26)
	HR	1	66.88 (64.14, 69.62)	25	73.04 (69.23, 76.84)	26	72.36 (68.82, 75.90)	25	6.16 (0.43, 11.89)	5.48 (0.01, 10.95)
		2	82.80 (79.01, 86.59)	25	70.00 (−)	1	78.09 (74.63, 81.56)	22	−12.8 (−17.43, −8.17)	−4.71 (−10.98, 1.57)
		3	82.75 (79.89, 85.61)	24	76.35 (72.70, 79.99)	26	82.63 (79.23, 86.02)	24	−6.40 (−12.06, −0.75)	−0.13 (−5.55, 5.30)
		4	81.28 (77.93, 84.63)	25	74.04 (70.20, 77.88)	25	79.38 (75.70, 83.05)	24	−7.24 (−13.46, −1.02)	−1.91 (−7.98, 4.17)
		5	78.46 (74.97, 81.95)	24	75.96 (71.47, 80.44)	24	81.58 (77.83, 85.33)	24	−2.50 (−9.44, 4.44)	3.13 (−3.13, 9.38)
		6	78.32 (75.04, 81.60)	25	73.89 (68.84, 78.93)	26	79.20 (76.52, 81.88)	25	−4.44 (−11.78, 2.91)	0.88 (−4.29, 6.05)
		7	79.32 (75.66, 82.98)	25	76.04 (70.85, 81.22)	26	79.76 (76.49, 83.04)	25	−3.28 (−11.03, 4.47)	0.44 (−5.56, 6.44)
		8	78.08 (74.44, 81.72)	24	74.46 (70.32, 78.60)	26	78.84 (75.89, 81.79)	25	−3.62 (−10.36, 3.11)	0.76 (−4.97, 6.48)
	SBP	1	125.32 (119.67, 130.97)	25	113.31 (109.9, 116.71)	26	114.88 (109.94, 119.83)	25	−12.01 (−18.61, −5.41)	−10.44 (−17.95, −2.93)
		2	122.68 (117.92, 127.44)	25	128 (−)	1	122.50 (120.34, 124.66)	22	5.32 (0.56, 10.08)	−0.18 (−5.41, 5.05)
		3	125.21 (121.00, 129.41)	24	118.73 (115.19, 122.27)	26	121.96 (119.65, 124.26)	24	−6.48 (−11.97, −0.98)	−3.25 (−8.04, 1.54)
		4	126.32 (122.36, 130.29)	25	122.20 (118.78, 125.62)	25	118.38 (115.95, 120.80)	24	−4.12 (−9.35, 1.11)	−7.95 (−12.59, −3.30)
		5	124.92 (120.71, 129.13)	25	118.88 (115.79, 121.96)	24	118.13 (115.65, 120.60)	24	−6.05 (−11.26, −0.83)	−6.80 (−11.67, −1.92)
		6	125.60 (120.79, 130.41)	25	120.60 (118.49, 122.71)	25	118.68 (116.83, 120.53)	25	−5.00 (−10.25, 0.25)	−6.92 (−12.08, −1.76)
		7	123.28 (119.67, 126.89)	25	121.62 (119.39, 123.84)	26	120.00 (117.32, 122.68)	25	−1.66 (−5.90, 2.58)	−3.28 (−7.77, 1.21)
		8	124.20 (119.97, 128.44)	25	121.58 (119.17, 123.99)	26	118.28 (115.40, 121.16)	25	−2.62 (−7.50, 2.25)	−5.92 (−11.04, −0.80)
	DBP	1	87.04 (77.73, 96.35)	25	74.08 (70.67, 77.48)	26	72.68 (69.06, 76.3)	25	−12.96 (−22.87, −3.05)	−14.36 (−24.35, −4.37)
		2	83.80 (80.56, 87.04)	25	-	0	84.21 (81.01, 87.42)	14	-	0.41 (−4.14, 4.97)
		3	85.30 (82.11, 88.50)	23	73.39 (71.07, 75.70)	26	83.05 (80.32, 85.78)	19	−11.92 (−15.87, −7.97)	−2.25 (−6.45, 1.95)
		4	81.80 (77.81, 85.79)	25	71.76 (69.29, 74.23)	25	76.08 (71.99, 80.18)	24	−10.04 (−14.74, −5.34)	−5.72 (−11.44, 0.00)
		5	84.40 (81.34, 87.46)	25	75.25 (71.15, 79.35)	24	81.50 (78.59, 84.41)	24	−9.15 (−14.27, −4.03)	−2.90 (−7.13, 1.33)
		6	83.36 (80.11, 86.61)	25	74.73 (72.05, 77.41)	26	85.12 (82.62, 87.62)	25	−8.63 (−12.84, −4.42)	1.76 (−2.34, 5.86)
		7	84.60 (80.66, 88.54)	25	77.85 (74.68, 81.01)	26	81.76 (79.07, 84.45)	25	−6.75 (−11.81, −1.70)	−2.84 (−7.61, 1.93)
		8	82.84 (79.94, 85.74)	25	74.12 (70.65, 77.59)	25	82.92 (80.16, 85.68)	25	−8.72 (−13.24, −4.20)	0.08 (−3.92, 4.08)
	MAP	1	99.80 (92.50, 107.10)	25	87.15 (84.13, 90.18)	26	86.75 (82.95, 90.54)	25	−12.65 (−20.55, −4.74)	−13.05 (−21.28, −4.83)
		2	96.76 (93.34, 100.18)	25	-	0	97.55 (95.07, 100.02)	14	-	0.79 (−3.43, 5.01)
		3	98.44 (95.25, 101.62)	23	88.50 (85.96, 91.04)	26	96.30 (94.07, 98.53)	19	−9.93 (−14.01, −5.86)	−2.14 (−6.02, 1.75)
		4	96.64 (92.86, 100.42)	25	88.57 (86.1, 91.04)	25	90.18 (86.87, 93.49)	24	−8.07 (−12.58, −3.55)	−6.46 (−11.48, −1.44)
		5	97.91 (94.64, 101.18)	25	89.79 (86.56, 93.02)	24	93.71 (91.27, 96.15)	24	−8.12 (−12.71, −3.52)	−4.20 (−8.28, −0.12)
		6	97.44 (94.07, 100.81)	25	90.01 (88.00, 92.03)	25	96.31 (94.37, 98.25)	25	−7.43 (−11.35, −3.50)	−1.13 (−5.02, 2.75)
		7	97.49 (93.97, 101.02)	25	92.44 (90.17, 94.7)	26	94.51 (92.21, 96.80)	25	−5.06 (−9.25, −0.86)	−2.99 (−7.20, 1.22)
		8	96.63 (93.52, 99.74)	25	89.85 (87.52, 92.19)	25	94.71 (92.38, 97.04)	25	−6.77 (−10.66, −2.88)	−1.92 (−5.81, 1.97)
Adjusted	Craving	1	1.63 (0.73, 2.53)	25	1.21 (0.50, 1.91)	26	0.70 (0.33, 1.07)	25	−0.42 (−1.90, 1.05)	−0.93 (−2.01, 0.14)
		2	1.79 (0.93, 2.65)	25	1.36 (0.42, 2.30)	26	0.90 (0.40, 1.40)	25	−0.43 (−2.05, 1.19)	−0.89 (−2.01, 0.22)
		3	1.55 (0.69, 2.41)	25	1.77 (0.76, 2.77)	25	0.98 (0.48, 1.48)	25	0.22 (−1.47, 1.91)	−0.57 (−1.68, 0.53)
		4	1.90 (0.85, 2.94)	22	2.77 (1.37, 4.16)	25	0.92 (0.37, 1.47)	23	0.87 (−1.31, 3.05)	−0.98 (−2.31, 0.36)
		5	1.91 (0.85, 2.97)	20	1.82 (0.63, 3.02)	25	0.93 (0.31, 1.55)	24	−0.09 (−2.10, 1.93)	−0.98 (−2.38, 0.42)
		6	1.23 (0.42, 2.04)	25	2.13 (0.84, 3.42)	26	0.62 (0.16, 1.08)	25	0.90 (−1.02, 2.82)	−0.61 (−1.62, 0.40)
		7	2.01 (0.91, 3.11)	20	1.82 (0.63, 3.01)	26	0.50 (0.10, 0.90)	25	−0.19 (−2.24, 1.86)	−1.51 (−2.85, −0.17)
		8	1.56 (0.64, 2.48)	20	3.09 (1.81, 4.37)	26	0.66 (0.17, 1.15)	25	1.53 (−0.47, 3.53)	−0.90 (−2.06, 0.25)
	Wellbeing	1	16.76 (15.00, 18.52)	25	18.23 (16.19, 20.26)	26	17.19 (16.61, 17.77)	25	1.47 (−1.84, 4.77)	0.43 (−1.82, 2.68)
		2	16.00 (14.30, 17.70)	25	19.23 (16.85, 21.61)	26	17.09 (16.52, 17.66)	25	3.23 (−0.38, 6.84)	1.09 (−1.11, 3.29)
		3	15.56 (13.80, 17.32)	25	15.92 (13.26, 18.58)	26	16.78 (16.17, 17.39)	25	0.36 (−3.55, 4.27)	1.22 (−1.06, 3.50)
		4	16.80 (15.00, 18.60)	25	19.94 (16.95, 22.93)	25	16.67 (16.06, 17.28)	24	3.14 (−1.19, 7.47)	−0.13 (−2.45, 2.19)
		5	16.44 (14.33, 18.55)	25	14.53 (11.71, 17.34)	25	16.87 (16.29, 17.44)	24	−1.91 (−6.25, 2.43)	0.43 (−2.24, 3.09)
		6	16.12 (14.05, 18.19)	25	14.42 (11.49, 17.35)	26	16.64 (16.04, 17.24)	25	−1.70 (−6.10, 2.69)	0.52 (−2.13, 3.16)
		7	16.08 (13.61, 18.55)	25	18.5 (15.71, 21.28)	26	16.54 (15.87, 17.21)	25	2.42 (−2.16, 6.99)	0.46 (−2.70, 3.63)
		8	16.56 (14.61, 18.51)	25	13.88 (11.38, 16.38)	26	16.71 (16.05, 17.36)	24	−2.68 (−6.58, 1.22)	0.15 (−2.36, 2.65)
	Sleep	1	14.57 (12.58, 16.56)	25	13.13 (11.06, 15.20)	26	12.80 (12.13, 13.47)	25	−1.44 (−4.94, 2.07)	−1.77 (−4.33, 0.79)
		2	13.17 (11.06, 15.28)	25	14.98 (12.90, 17.05)	26	12.87 (12.18, 13.56)	25	1.81 (−1.82, 5.44)	−0.30 (−3.00, 2.40)
		3	12.57 (10.99, 14.15)	25	10.75 (8.38, 13.12)	26	12.58 (11.87, 13.29)	25	−1.82 (−5.27, 1.63)	0.01 (−2.15, 2.17)
		4	11.53 (9.38, 13.68)	25	13.84 (10.55, 17.12)	25	12.24 (11.51, 12.96)	24	2.31 (−2.49, 7.10)	0.71 (−2.06, 3.47)
		5	12.77 (10.48, 15.06)	25	11.03 (8.14, 13.92)	25	12.58 (11.94, 13.23)	24	−1.74 (−6.25, 2.77)	−0.18 (−3.09, 2.72)
		6	11.61 (9.29, 13.93)	25	10.71 (8.15, 13.27)	26	12.25 (11.55, 12.95)	25	−0.90 (−5.12, 3.32)	0.64 (−2.31, 3.60)
		7	11.13 (8.76, 13.50)	25	10.63 (7.60, 13.66)	26	12.22 (11.50, 12.94)	25	−0.50 (−5.17, 4.18)	1.09 (−2.01, 4.18)
		8	11.89 (9.36, 14.42)	25	10.13 (7.63, 12.63)	26	12.26 (11.56, 12.97)	24	−1.76 (−6.08, 2.57)	0.37 (−2.88, 3.63)
	HR	1	67.67 (65.24, 70.10)	25	73.84 (70.73, 76.95)	26	74.43 (72.71, 76.14)	25	6.17 (1.42, 10.92)	6.76 (3.15, 10.37)
		2	83.59 (80.47, 86.71)	25	73.37 (−)	1	77.00 (75.36, 78.63)	22	−10.22 (−14.17, −6.27)	−6.59 (−10.93, −2.25)
		3	83.18 (79.66, 86.70)	24	77.15 (74.29, 80.01)	26	79.03 (77.14, 80.93)	24	−6.03 (−11.59, −0.47)	−4.14 (−8.91, 0.62)
		4	82.07 (78.79, 85.35)	25	74.87 (71.21, 78.53)	25	77.58 (75.88, 79.27)	24	−7.20 (−13.22, −1.17)	−4.49 (−8.9, −0.08)
		5	79.44 (76.26, 82.62)	24	76.82 (73.59, 80.05)	24	78.57 (76.64, 80.49)	24	−2.61 (−8.07, 2.85)	−0.87 (−5.37, 3.63)
		6	79.11 (75.63, 82.59)	25	74.69 (69.44, 79.93)	26	77.50 (76.16, 78.84)	25	−4.42 (−12.1, 3.26)	−1.61 (−6.18, 2.95)
		7	80.11 (76.88, 83.33)	25	76.84 (71.48, 82.20)	26	77.75 (76.16, 79.34)	25	−3.27 (−10.93, 4.40)	−2.36 (−6.67, 1.95)
		8	79.06 (75.34, 82.78)	24	75.26 (71.27, 79.26)	26	77.33 (75.92, 78.75)	25	−3.80 (−10.46, 2.86)	−1.73 (−6.55, 3.09)
	SBP	1	123.55 (120.19, 126.92)	25	116.09 (112.74, 119.43)	26	116.34 (114.13, 118.55)	25	−7.47 (−13.27, −1.66)	−7.22 (−12.13, −2.31)
		2	120.91 (117.43, 124.40)	25	124.39 (−)	1	119.72 (118.59, 120.85)	22	3.47 (−0.96, 7.91)	−1.19 (−5.72, 3.34)
		3	123.17 (120.22, 126.12)	24	121.51 (118.18, 124.84)	26	119.48 (118.37, 120.59)	24	−1.66 (−7.08, 3.76)	−3.69 (−7.55, 0.17)
		4	124.55 (121.28, 127.83)	25	125.04 (121.12, 128.95)	25	117.89 (116.81, 118.96)	24	0.48 (−5.81, 6.78)	−6.66 (−10.87, −2.45)
		5	123.15 (119.9, 126.41)	25	122.05 (118.81, 125.28)	24	117.78 (116.68, 118.88)	24	−1.10 (−6.77, 4.56)	−5.37 (−9.56, −1.19)
		6	123.83 (120.57, 127.10)	25	123.64 (120.76, 126.51)	25	118.02 (117.19, 118.86)	25	−0.20 (−5.36, 4.96)	−5.81 (−9.97, −1.64)
		7	121.51 (118.93, 124.09)	25	124.40 (121.75, 127.04)	26	119.26 (117.75, 120.76)	25	2.88 (−1.60, 7.37)	−2.26 (−5.93, 1.41)
		8	122.43 (119.24, 125.62)	25	124.36 (121.32, 127.40)	26	117.85 (116.57, 119.13)	25	1.92 (−3.49, 7.34)	−4.59 (−8.78, −0.39)
	DBP	1	86.66 (77.57, 95.74)	25	74.67 (71.29, 78.05)	26	74.27 (71.05, 77.49)	25	−11.98 (−23.88, −0.09)	−12.39 (−24.23, −0.55)
		2	83.42 (80.48, 86.35)	25	-	0	83.31 (80.62, 85.99)	14	-	−0.11 (−4.00, 3.79)
		3	84.90 (82.11, 87.70)	23	73.98 (71.40, 76.55)	26	82.40 (80.15, 84.64)	19	−10.93 (−15.75, −6.1)	−2.51 (−6.81, 1.80)
		4	81.42 (77.69, 85.14)	25	72.34 (69.08, 75.61)	25	76.93 (73.62, 80.25)	24	−9.07 (−15.36, −2.78)	−4.48 (−10.67, 1.71)
		5	84.02 (81.24, 86.79)	25	75.88 (71.49, 80.27)	24	81.18 (78.87, 83.49)	24	−8.14 (−14.63, −1.65)	−2.84 (−7.20, 1.53)
		6	82.98 (80.04, 85.91)	25	75.33 (72.65, 78.01)	26	84.02 (81.76, 86.28)	25	−7.65 (−12.66, −2.63)	1.04 (−3.32, 5.40)
		7	84.22 (80.50, 87.93)	25	78.44 (74.26, 82.63)	26	81.13 (78.89, 83.38)	25	−5.77 (−12.82, 1.27)	−3.08 (−8.32, 2.16)
		8	82.46 (79.76, 85.15)	25	74.80 (70.56, 79.04)	25	82.29 (80.02, 84.56)	25	−7.66 (−14.02, −1.29)	−0.16 (−4.36, 4.03)
	MAP	1	99.09 (92.36, 105.82)	25	88.22 (84.94, 91.51)	26	88.52 (85.61, 91.43)	25	−10.87 (−20.11, −1.63)	−10.58 (−19.58, −1.58)
		2	96.05 (93.24, 98.87)	25	-	0	95.98 (93.92, 98.04)	14	-	−0.07 (−3.45, 3.30)
		3	97.70 (95.26, 100.15)	23	89.57 (86.94, 92.20)	26	95.12 (93.36, 96.87)	19	−8.13 (−12.75, −3.52)	−2.59 (−6.13, 0.96)
		4	95.93 (92.60, 99.27)	25	89.64 (86.19, 93.10)	25	90.89 (88.55, 93.24)	24	−6.29 (−12.49, −0.09)	−5.04 (−10.11, 0.03)
		5	97.20 (94.47, 99.93)	25	90.97 (87.24, 94.70)	24	93.33 (91.62, 95.04)	24	−6.24 (−12.11, −0.36)	−3.87 (−7.76, 0.01)
		6	96.73 (94.00, 99.47)	25	91.21 (88.63, 93.80)	25	95.12 (93.52, 96.72)	25	−5.52 (−10.37, −0.67)	−1.61 (−5.35, 2.13)
		7	96.79 (93.72, 99.86)	25	93.51 (89.92, 97.09)	26	93.76 (92.04, 95.47)	25	−3.28 (−9.34, 2.77)	−3.03 (−7.26, 1.20)
		8	95.92 (93.22, 98.62)	25	91.05 (87.44, 94.66)	25	94.02 (92.32, 95.72)	25	−4.87 (−10.71, 0.97)	−1.90 (−5.70, 1.89)

The adjusted model was adjusted for baseline assessments. C, control group; CC, Chan-Chuang Group; R, resistance group. MD, mean difference. CI, confidence interval. N indicates the sample size employed for corresponding analysis; The pairwise comparisons use the control group as a referent.

### 3.4. Retention and adherence to program

**Figure 2:**
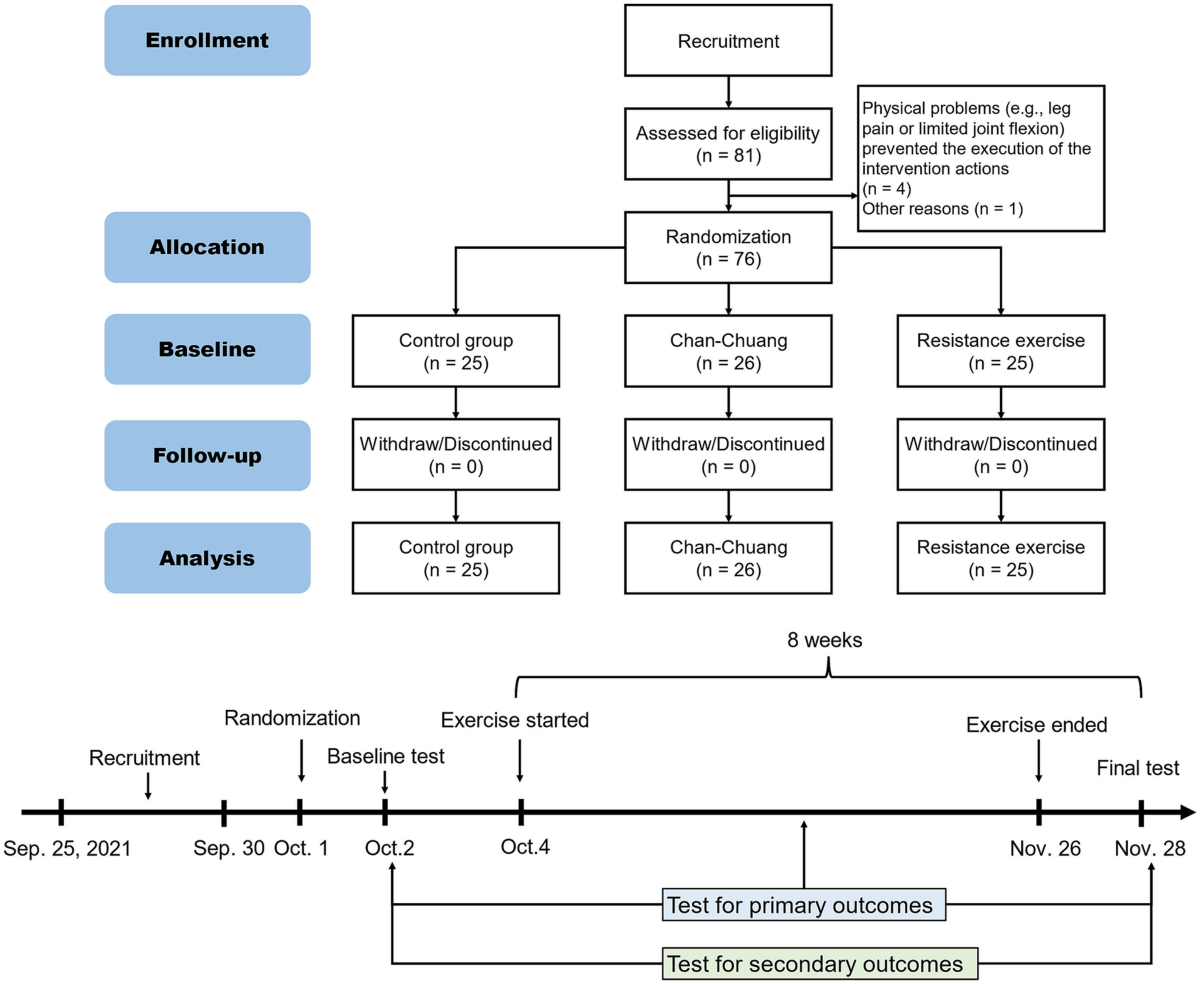
The experimental procedure and timeline.

A total of 81 people were initially recruited and 76 qualified ones were included in randomization and the following intervention. Data were analyzed according to the original experimental groups, with the sample sizes/observations used as shown in Tables 1, 2. Some participants were absent for one or several tests for quarantine or personal reasons, but all the participants were eligible for analysis as they had completed the intervention and met our inclusion criteria (Figure 2). No participants dropped out of the intervention due to unintended harm or other adverse events. The recruitment was started on September 25, 2021, and ended on September 30 (Figure 2). The whole experiment ended on November 28, 2021, as planned.

### 3.5. Sensitivity analysis

In our sensitivity analysis, we did not observe any substantial change regarding the effects of the two exercises when compared to the control group. However, after adjusting for participants’ characteristics (including age, drug use history, and detention duration), the drug craving level of the resistance exercise group was significantly lower than that of the Chan-Chuang group (Supplementary Table S4).

## 4. Discussion

Our study aimed to examine the effects of Chan-Chuang and resistance exercise on drug rehabilitation. There were some inter-group differences in our primary indicators at baseline, which may imply inadequacies in the effectiveness of randomization. Therefore, we primarily focus on and discuss the results after baseline adjustment. We did not find a distinct effect on drug craving, mental wellbeing, and sleep quality. Nevertheless, we found that Chan-Chuang had positive effects on HR, DBP, MAP, vital capacity, grip strength, and balance. Resistance exercise showed positive effects on SBP, MAP, vital capacity, grip strength, and vertical jump.

### 4.1. Chan-Chuang exercise

Regarding our primary outcomes, we did not observe any positive change in drug craving, mental wellbeing, and sleep quality. Since this is the first study on this exercise type and methamphetamine rehabilitation, we are not able to compare our results with those of other studies. From the perspective of mindfulness, these null effects are not surprising, because the known effects of mindfulness-based interventions on drug craving and mental outcomes are still controversial (32–34). The null effects, according to our assumptions, could be due to Chan-Chuang’s insufficient physiological stress. Dopamine response is essential to change drug cravings and emotional outcomes. However, Chan-Chuang may be too mild to induce sufficient dopamine response.

On the bright side, we found that Chan-Chuang generally improved HR, DBP, and MAP. The decreased HR may echo previous studies on isometric squat exercise and mindfulness (35, 36). A meta-analysis has revealed that isometric exercise can lower SBP, DBP, and MAP more effectively than dynamic aerobic or resistance exercise (37). Likewise, we also observed a greater reduction in DBP in the Chan-Chuang group than in the resistance exercise group, which may support the advantage of isometric exercise in blood pressure regulation.

Regarding our secondary outcomes, we found Chan-Chuang improved vital capacity, grip strength, and balance. It is suggested that respiratory functions are associated with isometric muscular strength (38). Chan-Chaung can practice isometric muscular functions, which may explain the greater improvement in Chan-Chuang than in the resistance exercise group.

The findings on grip strength and balance are in line with some studies on college students and athletes (39, 40). Although the underlying mechanism is understudied, these findings may collectively support the general benefits of the Chan-Chuang exercise.

### 4.2. Resistance exercise

Regarding our primary outcomes, we did not observe any positive effect of resistance exercise on drug craving, mental wellbeing, and sleep quality. These results were inconsistent with a previous study where 12 weeks of resistance training positively changed anxiety, depression, sleep quality, and drug craving among Chinese methamphetamine users (41). These discrepancies may come from different study designs. The previous study used a self-control design and only pre-post comparisons were made. Hence, the effects of routine rehabilitation and human seasonal response were not ruled out (42, 43). By comparison, we employed a randomized controlled design, which may challenge the reported positive effects of resistance exercise on drug craving. On the other hand, we found resistance exercise generally reduced SBP and MAP. These findings reinforce previous studies that resistance training can improve cardiovascular fitness (44, 45).

Regarding our secondary outcomes, we found significant improvements in vital capacity, grip strength, and vertical jump. A relevant study has reported that resistance exercise might enhance the maximum ventilation volume of drug addicts, indicating a benefit for lung functions (46). This study is somewhat supported by our enhanced vital capacity. The increased grip strength and vertical jump may, to a certain extent, prove the role of resistance exercise in proving muscular strength. Thereby, these findings may somewhat support a relevant eight-week intervention in which resistance and aerobic exercise increased methamphetamine users’ leg press and chest press strength (47). These benefits are similar to the known effects of resistance exercise on the general public (48, 49), again confirming the promotion of the fitness function of resistance exercise.

Generally, our isometric exercise (Chan-Chuang) and resistance exercise using only elastic bands have very low requirements for both the environment and equipment, making them a viable option for home-based workouts during the pandemic (50). Therefore, these exercises may have considerable applicability within the incarcerated population, particularly when considering the large number of incarcerated individuals in Chinese detention facilities and the relatively limited number of management personnel.

### 4.3. Limitation

The power calculation in our study was conducted after participant enrollment but before the intervention. It revealed satisfactory statistical power for primary outcomes but very low power for secondary outcomes. This quasi *post hoc* power calculation may have reduced our ability to detect effects, increased the type I error rate, and limited the generalizability of our findings. Furthermore, the fear of stigma may lead to a biased sample of participants, with those who are more comfortable discussing their drug use or have less to lose being overrepresented. Participants might also underreport their drug cravings due to the fear of judgment or negative consequences, which can lead to inaccurate data and underestimate the true extent of drug cravings. We observed some differences in our outcome variables at the baseline. Although baseline differences do not necessarily indicate an unsuccessful randomization (51), they might influence our estimation. These disparities may stem from our use of a relatively small sample size (52), which necessitates future re-examinations.

## 5. Conclusion

This study aimed to examine the effectiveness of Chan-Chuang and resistance exercise on drug rehabilitation of methamphetamine users at a Chinese mandatory detention center. Although we did not observe positive results for drug craving, mental wellbeing, and sleep quality, we found both Chan-Chuang and resistance exercises lowered blood pressure and improved physical fitness. Since methamphetamine users usually have poor physical health conditions, our findings may indicate the positive roles of resistance and Chan-Chuang exercise in drug rehabilitation at Chinese mandatory detention centers.

## Data availability statement

The raw data supporting the conclusions of this article will be made available by the authors, without undue reservation.

## Ethics statement

This study involving human participants was reviewed and approved by the Institutional Review Board of the College of Physical Education of Southwest University (code: 2021307009). The study was registered via the Chinese Clinical Trial Registry (code: ChiCTR2100049985). Participation in this study was purely voluntary. We introduced our plan and distributed recruitment messages to the detainee through routine meetings at the correctional center. Written informed consent to participate in this study was provided by our participants.

## Author contributions

HL: conceptualization, investigation, and writing – original draft. CW, XH, and LX: investigation. YC: writing – review & editing. YL: funding acquisition. GZ: supervision, project administration, and review & editing. All authors contributed to the article and approved the submitted version.
