# Supplementary material for: Chan-Chuang and resistance exercise for drug rehabilitation: a randomized controlled trial among Chinese male methamphetamine users
Source: Front Public Health. 2023 Oct 26;11:1180503. doi: 10.3389/fpubh.2023.1180503 (PMC10642185; doi:10.3389/fpubh.2023.1180503)
Supplement: Supplementary file 1 [file Table_1.DOCX]

**研究知情同意书**

**研究名称：**站桩对戒毒人员健康促进功效的初步研究

**主要研究者：**张国栋，李汉森

**申办方**：西南大学

**尊敬的受试者：**

您被邀请参加站桩对戒毒人员健康促进功效的初步研究。请仔细阅读同意书并慎重做出是否参与本研究的决定。参与这项研究完全是您的自主选择。作为受试者，您必须再加入研究前向工作人员出示您的同意书，您可以请工作人员给您解释不明白的地方。您有权拒绝参与本研究，也可以随时退出该研究，且不会受到处罚，也不会失去应有的权利。本研究的背景、目的，和过程如下：

**1.研究背景**

站桩是中国的传统功法，也是疫情期间本市推崇的居家健身方式。诸多研究与经验表明，站桩可以促进人的心理健康并维持骨骼肌系统的正常运转，尤其对体弱者具有特别的功效。然而，站桩是否适合服务于戒毒人员的健康保健还不清楚。本研究旨在使用站桩作为运动干预措施，初步探寻此传统功法对于接受戒毒教育人员的健康促进价值。

**2. 研究目的**

探究站桩对戒毒人员的健康促进功能。

**3. 研究步骤**

于2021年中旬设计研究流程。招募信息由西南大学学生招募团队在重庆教育矫正中心的内部会议上进行推送。受试者限定为具备基础身体活动能力的目前在教育矫正中心接受教育的有甲基苯丙胺使用史的人员。未满18岁者不得参与。

**4.风险与收益**

参与运动干预具有一定风险，例如可能造成肌肉骨骼反应，包括但不限于关节不适，肌肉酸痛，心理疲劳等。同时，参与此干预可能具有一些收益。基于一般运动干预的反馈，参与此干预可能有助于强健运动系统，改善劳动工作能力并提高心血管等身体内部功能。此外，参与此干预可能助力于未来为本教育矫正中心开发多样化的业余活动内容。

**5.研究结果使用与个人信息保密**

本研究结果可能会在医学杂志上发表。但我们会按照法律要求为您保密研究记录。受试者信息将受到严格保密。必要时，政府管理部门和伦理道德委员会可能按规定查阅您的资料。

**6.费用及补偿**

参与本研究可以获得一定的经济补偿或奖励。

**7.受试者权利和注意事项**

整个实验过程您都是自愿参加的。如果您决定不参与本研究，不会对您的其他权益造成影响。如果您自愿参加，请在知情同意书上签字。您有权在试验的任何阶段退出并不会受到任何负面影响。

**受试者签字页**

**知情同意声明：**

我已被告知此研究的背景、目的、过程、风险和收益等情况。我有足够的时间进行询问，并得到了满意的答复。

我已阅读这份知情同意书，并且愿意参加本研究。

我知道我可以选择不参加本研究，并且可以在研究任何阶段无需理由退出。

我如果遭遇不良后果或事件，或者研究人员认为我继续参加将不符合我的最佳利益，他/她会决定让我退出研究，无需征得我的同意。

受试者签字： 日期：

（注：如果受试者无行为能力/限制行为能力时，则需要法定代理人签名和签署日期）
