# Supplementary material for: Chan-Chuang and resistance exercise for drug rehabilitation: a randomized controlled trial among Chinese male methamphetamine users
Source: Front Public Health. 2023 Oct 26;11:1180503. doi: 10.3389/fpubh.2023.1180503 (PMC10642185; doi:10.3389/fpubh.2023.1180503)
Supplement: Supplementary file 2 [file Table_2.DOCX]

**Research Informed Consent Form**

**Study Title:** A Preliminary Study on the Health Promotion Effects of Chan-Chuang for Drug Rehabilitation Patients

**Principal Investigators:** Guodong Zhang, Hanshen Li

**Sponsor:** Southwest University

**Dear Participant:**

You are invited to participate in a preliminary study on the health promotion effects of Chan-Chuang for drug rehabilitation patients. Please carefully read this informed consent form and make a thoughtful decision on whether or not to participate in this study. Participation in this study is entirely voluntary. As a participant, you must present this consent form to the study staff before joining, and you can ask the staff to explain anything you do not understand. You have the right to refuse to participate in this study, and you can withdraw from the study at any time without penalty or loss of your rights. The background, objectives, and procedures of this study are as follows:

**Background**

Chan-Chuang is a traditional Chinese practice and a recommended home exercise during the pandemic. Numerous studies and experiences have shown that Chan-Chuang can promote mental health and maintain the normal functioning of the musculoskeletal system, especially for the weak-bodied. However, it is unclear whether Chan-Chuang is suitable for health care for drug rehabilitation patients. This study aims to use Chan-Chuang as an exercise intervention measure to explore the health promotion value of this traditional practice for drug rehabilitation patients.

**Objectives**

To investigate the health promotion function of Chan-Chuang for drug rehabilitation patients.

**Study Procedures**

The study process was designed in mid-2021. The recruitment information was pushed by the Southwest University student recruitment team at an internal meeting of the Chongqing Education Correction Center. The subjects were limited to individuals with basic physical activity ability who are currently receiving education in the correctional center and have a history of methamphetamine use. Individuals under 18 years of age are not allowed to participate.

**Risks and Benefits**

Participating in exercise intervention has some risks, such as possible musculoskeletal reactions, including but not limited to joint discomfort, muscle soreness, and psychological fatigue. At the same time, participating in this intervention may have some benefits. Based on feedback from general exercise interventions, participation in this intervention may help to strengthen the exercise system, improve work capacity, and enhance the internal functions of the cardiovascular system and other body parts. In addition, participating in this intervention may help to develop diversified amateur activities for this correctional center in the future.

**Use of Research Results and Confidentiality of Personal Information**

The results of this study may be published in medical journals. However, we will keep your research records confidential as required by law. The subject information will be kept strictly confidential. If necessary, government regulatory agencies and ethical committees may review your information according to regulations.

**Costs and Compensation**

Participation in this study can receive some economic compensation or rewards.

**Participant Rights and Precautions**

You are volunteering for the entire experimental process. If you decide not to participate in this study, it will not affect your other rights. If you voluntarily participate, please sign on the informed consent form. You have the right to withdraw at any stage of the trial and will not be negatively affected.

**Signature page for participants**

**Informed Consent Statement:**

I have been informed of the background, objectives, procedures, risks, and benefits of this study. I have sufficient time for inquiries and have received satisfactory answers. I understand that participation in this study is voluntary, and I am free to withdraw at any time. I agree to participate in this study.

Participant Signature: ______________________

Date: _______________
