# Supplementary figures and images for: Chan-Chuang and resistance exercise for drug rehabilitation: a randomized controlled trial among Chinese male methamphetamine users
Source: Front Public Health. 2023 Oct 26;11:1180503. doi: 10.3389/fpubh.2023.1180503 (PMC10642185; doi:10.3389/fpubh.2023.1180503)

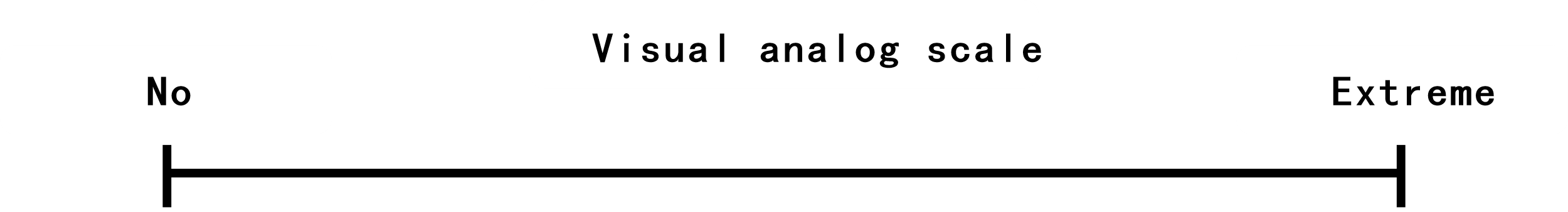


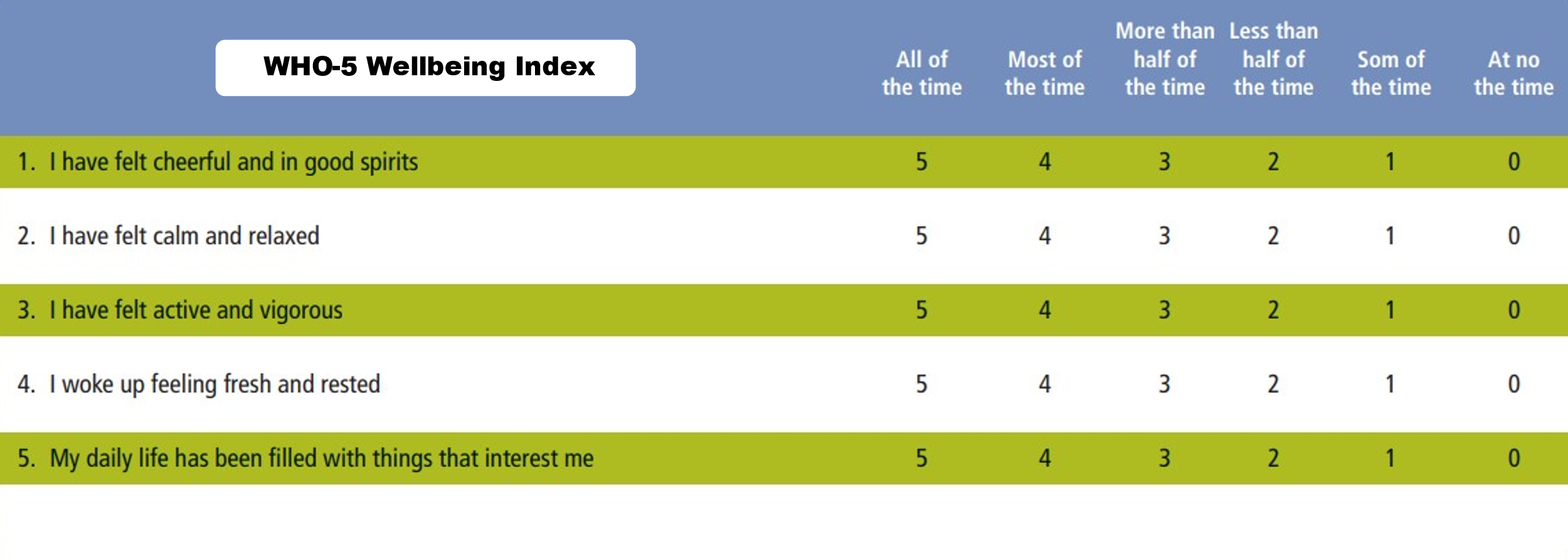


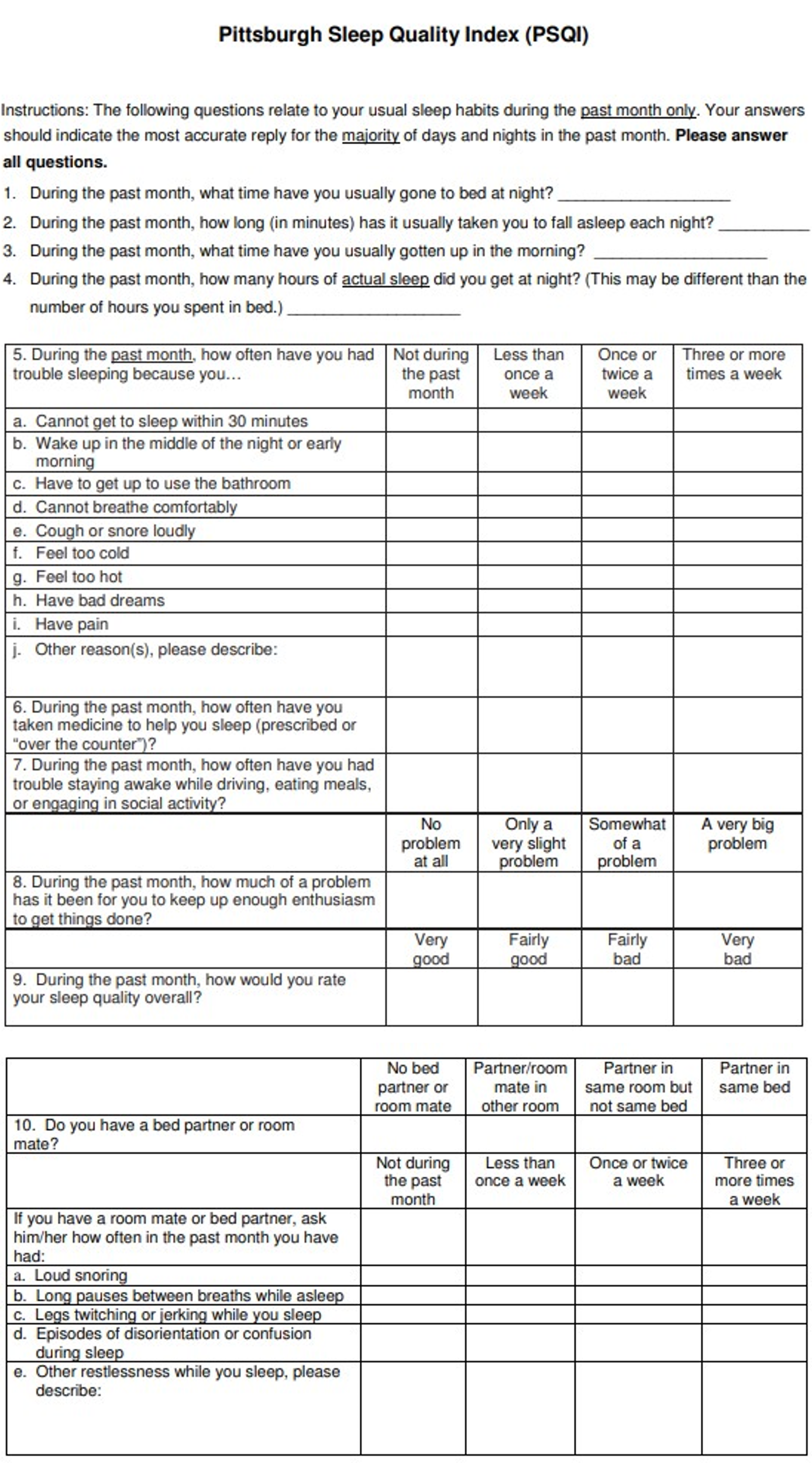

Supplement: Supplementary file 3 [file Table_3.DOCX]
